# Augustin Prichard

**Published:** 1898-03

**Authors:** 


### The Bristol Medico-Chirurgical Journal.

MARCH, 1898.

### In Memoriam.

### AUGUSTIN PRICHARD,

F.R.C.S. Eng., M.D. Berlin, L.S.A.

In the first decade of the present century, Bristol had the good fortune to be selected as the future residence of a promising young physician of extraordinary ability. Endowed with talents of the highest and most varied order, with a most retentive memory, and with indomitable perseverance, he was determined to devote to the profession of his choice the great ability and energy which he possessed. He began well when he took his degree at Edinburgh; for the subject of his thesis was, "De generis humani varietate." He had been educated at a first-rate school, where he had been thoroughly grounded in ancient languages, literature, and history. After leaving school he had, by continental travel, acquired an equally good knowledge of modern languages and of the natural sciences which particularly bore on the great subject of his Edinburgh thesis. In addition he was, by industry and close study, laying the foundations of his well-known work, *The Physical History of Man*,[1] which he published some years afterwards, and which gained him in England the honoured title of F.R.S., and abroad the still greater honour of a European reputation, for his book soon became a classic, and was acknowledged to be the best book of the time on anthropology. This scholarly and learned man was Dr. James Cowles Prichard, the father of Augustin Prichard, who became such a distinguished practitioner and teacher of surgery in our midst, and whose death we and all Bristol have recently had to lament.

Augustin Prichard, the second son of Dr. J. C. Prichard, was born at 39 College Green, on July 16th, 1818. After being for some time at a school which is described in the local directory of the period as a "French and Commercial School," and which had been from 1824 under the direction of Mr. F. Norton, at 33 Old Park, Bristol, he went to the Bristol College at the age of 13, and remained there for three years, when he was apprenticed to his uncle, Mr. J. B. Estlin, a well-known surgeon of the time. During this time he studied at the Bristol Infirmary and Medical School. In 1839 he became a student at St. Bartholomew's Hospital, and took the M.R.C.S. and L.S.A. in the following year. After this he went to Berlin, where he took his M.D. From Mr. Estlin, who was a distinguished oculist, Mr. Prichard had derived a taste for ophthalmology, and the subject of his thesis at Berlin was "Iritis." From Berlin he went to Vienna, and had the advantage of seeing the eye work of Jaeger. He came back

[1] Professor E. B. Poulton, F.R.S., writing in *Science Progress* in April, 1897, on "A Remarkable Anticipation of Modern Views on Evolution," says: "Prichard apprehended with perfect clearness that domesticated races of animals and plants have been produced by the selection of man and not by favourable surroundings, careful training or cultivation. He believed in the possibility of organic evolution and supported it by excellent arguments which still have the strongest weight to-day. He even recognised the operation of natural selection although he assigned to it a subordinate *rôle*. The most important anticipation is, however, the masterly discussion on the transmission of acquired characters, a discussion in which the distinction between acquired and inherent or congenital characters is clearly drawn, and many of the most difficult cases are fully argued out, the conclusions reached being those independently arrived at by Professor Weismann over half a century later."

to England in 1841, but soon after went to Paris for the purpose of attending lectures there. In October, 1842, he began practice at first in lodgings in College Green, and a year or two afterwards at his father's house, "The Red Lodge," in Park Row. In 1845 he married Miss Mary Ley, who died about five years ago. Four sons and three daughters survive him.

Mr. Prichard was appointed lecturer on Anatomy in the Bristol School in 1843. The museum of the School still contains some preparations which testify to his skill and industry. He became F.R.C.S. in 1849, and in the next year he was elected Surgeon to the Infirmary, a post which he held with much honour for twenty years, when, by the rule of the institution, he was obliged to retire. During the last five years of his lectureship on Anatomy he lectured also on Surgery at the School, work which he began in 1849 and continued till 1864. From 1844 to 1854 he was also Honorary Secretary to the School. For a long period he was Surgeon to the Bristol Eye Dispensary.

The British Medical Association found in Mr. Prichard an active member. Twice at the annual meetings—at Swansea in 1853 and at Bristol in 1863—he was the Surgical Orator. In 1857 he was the President of the Bath and Bristol Branch. When the Association met at Worcester in 1882 he was President of the Surgical section.

Mr. Prichard was greatly interested in the Bristol Medico-Chirurgical Society, which was formed in 1874, and in the session of 1877-78 he occupied the Presidential chair. Its *Journal* received from him several signed and unsigned contributions.

All through life Mr. Prichard was extremely fond of sketching, and had made a large number of water-colours, mostly representing scenes in old Bristol. Even till quite lately he was in the habit at Christmas of sending to many friends card sketches done by himself, and these were always specially valued.

After a long life of singularly good health he was taken on December 11th with symptoms of acute intestinal obstruction, and his condition rapidly became so serious that an operation had to be undertaken for its relief. The abdomen was opened and an attempt made to find the cause of obstruction. Owing to the extreme distension of the small intestines nothing could be discovered, so an artificial anus was made by fixing a piece of small intestine to the abdominal incision. This was opened after twenty-four hours and gave great relief to all the symptoms, so much so, that in a few days the obstruction, which was probably due to a volvulus, gave way and normal rectal defæcation again became established. The fæcal fistula gave rise to so much irritation and exhaustion, that an operation for its closure was performed two days before his death. This was followed by a recurrence of the original symptoms, and he gradually sank, dying peaceably at midnight on January 5th. In addition to his two sons, Dr. E. Long Fox and Mr. E. C. Board were in constant attendance, while Mr. W. H. Harsant, assisted by Dr. James Swain, performed the operations.

The noble example of uprightness and devotion to work which he has left behind is well described by some of those who were intimately associated with him for long periods, and whose communications which we print below will, we are sure, be read with interest and with profit.

Dr. J. G. Swayne writes:

"I am able to give some recollections of Augustin Prichard's early life which may be of interest. He and I were school-fellows at a new College, the first of its kind in Bristol, which was opened with much *éclat* in 1831. This College was domiciled in a spacious old mansion in Park Row just opposite a venerable house of Elizabethan fame, the 'Red Lodge,' in which Dr. J. C. Prichard lived.

"The urgent need of a higher grade of school in Bristol had long been felt, and the result was that several Bristol citizens did their best to supply the deficiency. It is much to the credit of our profession that several members of it took a leading part in this movement. Amongst them were Dr. J. C. Prichard, then the Senior Physician at the Infirmary; Dr. Andrew Carrick, a canny Scot, who had held the same post and had done a good Clifton practice before the end of last century; my father, Mr. J. C. Swayne, a professional friend of Dr. Prichard, both of whom were born in 1786; and Dr. Symonds, a rising young physician of much promise, who had lately come to Clifton.

"The masters appointed to the Bristol College were well chosen. Dr. Jerrard, the Principal, was a Fellow of Caius, Cambridge, and an excellent classical scholar. Dr. Butterton, of St. John's, Cambridge, the Vice-Principal, was equally strong in mathematics and was also a good classic. Dr. Butterton was the first to leave. When appointed head-master of a school in the north, he was succeeded by the Rev. J. E. Bromby, whose brother after having been Bishop of Tasmania for many years is now living in Clifton. When Dr. Jerrard followed some little time after, having been made Classical Examiner to the University of London, the managers secured for the classical teaching a scholar of first-rate ability in the person of Francis W. Newman, the brother of John Henry Newman, and he proved to be the best classical tutor we ever had. He did his work so well that he was in a great measure the cause of the extraordinary success of several of the older College pupils at Oxford.

"The year 1831 was a memorable, or rather notorious, one for then the Reform mania was waxing strong and brought a host of troubles to Bristol and its respected Mayor, Mr. Charles Pinney. The unsettled state of affairs acted prejudicially against the newly-born College. It made the moneyed classes shy of investing capital in a new undertaking of the kind. When the expected scholars were shown into a large room to be received by the masters, less than forty boys appeared. This was a poor beginning. At that time I was just over 11 years of age, and was one of the smallest boys present. The biggest, who was a tall pale youth with rather red hair, was Dr. J. C. Prichard's eldest son, James. Dr. Jerrard used to coach him in a room apart from the other boys, for he was there for a special purpose, as he was going to Oxford to try for a scholarship. About two months after this, James Prichard received the warmest congratulations of the Council, masters, and scholars of the Bristol College, for he had gained a good scholarship at Oriel. This was the beginning of his Oxford career, which was one uninterrupted success. He afterwards gained an open Oriel Fellowship and, at his final examination, a first class in Classics and the prize poem for Latin verse. After leaving Oxford he took Holy Orders, and entered upon his clerical duties in places remote from this city, and so I saw but little of him during the rest of his life. He died of consumption in 1848.

"Dr. Prichard's second son, Augustin, was many years younger than his brother James. He was about four months younger than my eldest brother George. I knew him better than any other member of Dr. Prichard's family. After being my fellow-pupil, he was during a long life a very true friend, professional ally, and colleague as lecturer. My brother George and he and I were all present at the opening of the College. During the later years of my school life I saw little of Augustin Prichard, for his father, wishing him to begin his medical education early, especially in the surgical branch of the profession, apprenticed him in 1834, when he was only 16, to his uncle, Mr. J. B. Estlin, a very clever surgeon and oculist. Augustin never showed the same amount of talent for classical literature as his brother James and his younger brother Constantine, and therefore he was not sent to Oxford or Cambridge, neither of which at that time was a very good place for medical and surgical instruction.

"Constantine, the next brother, who was three or four months my junior, and Samuel William Wayte, who is, I think, a few days younger than I am, and who is now living in Clifton, were still at the College when I left at the age of 17. For some time before I left I was in the same class (the first classical) with Constantine and Wayte, but never managed to get ahead of either of them. I had changed places two or three times with Augustin. I found all three most pleasant companions. They were model boys, and nearly always earned the best report of conduct which the Principal could give. Constantine Prichard and Samuel Wayte had been for some time at the top of the first class in classics—the head boys of the College—and were nearly always equal. Constantine besides being very good at his books was very good at play, and was the best cricketer in the College Club. They were both reading hard, and soon went to Oxford to try for a scholarship at Balliol. This was won by

Constantine, but Wayte consoled himself by gaining a scholarship at Trinity. When they went up for the final examination, Constantine took a first class in classics and gained the English Prize Essay, but Wayte outstripped his former opponent by taking a double first. He told me that he owed his success in mathematics chiefly to the coaching he had received from the Rev. J. E. Bromby. Wayte became a Fellow of Trinity and settled down into the quiet life of a College Don and Fellow Residentiary, enjoying his *otium cum dignitate* till he attained the higher honour of becoming one of the heads of houses about 1867, when he was elected President of Trinity. This office he held for rather more than ten years, when he resigned and came to Clifton in order to be nearer to his father, who was now well advanced in years. Constantine Prichard, a few years after taking his degree, was ordained and had a living in Rutlandshire. In 1869 he died from the same complaint that had proved fatal to his brother James, and left a widow and family.

"The details of Augustin's movements immediately after he left school are admirably given by himself in a clever and amusing little book which, under the title of *A few Medical and Surgical Reminiscences*, he published about two years ago, and of which a sequel was in the press at the time of his death.

"Of his younger brothers I am not able to say much. Theodore went to Oxford after being at the same school, but he had scarcely finished his career there, taking a second class in classics, when he died at the age of 24. Iltudus became an officer in the Indian Army, and was present at the battles of Chillianwallah and Gujerat. After the Mutiny was over he and his younger brother Albert became joint editors of an Indian newspaper. Iltudus died in 1874, and Albert came back to England, and is now living in the neighbourhood of London.

"As I have said so much about the school at which I received my early education, it would be unpardonable if I omitted to mention the coming of a stout muscular youth with a florid complexion, who was a perfect mathematical genius, and who is now known as Sir George Gabriel Stokes, Bart., F.R.S., D.C.L., LL.D. He was born August 13th, 1819, and educated first at a school in Dublin. After he left the Bristol College he went to Cambridge, where he was Senior Wrangler in 1841. In 1869 he was President of the British Association, and on the retirement of Prof. Huxley in 1885 he became President of the Royal Society. In 1887 he was elected M.P. for Cambridge University.

"I am proud of having been educated at a school that could send to Oxford and Cambridge men who have been so crowned with honours as the Prichards, Wayte, and Stokes. And yet the College that produced such marvellous results lived only about 11 years. Dr. Gray, the much respected Bishop of Bristol, and many of the clergy, were opposed to it on the ground that the sons of Nonconformists were allowed to attend the School and not receive the religious instruction provided for Churchmen. In *The Annals of Bristol in the Nineteenth Century* Mr. Latimer well sums up the history of this Bristol College, and of the Bishop's College, which was intended to supersede it.

"It will have been seen from what I have said that Augustin Prichard belonged to a family intellectually far above the average. His father's erudition impressed me at my first interview with him, when I was about 15 years of age. My grandfather, Dr. Griffiths, had retired from practice and was living at Westbury. One Saturday half-holiday when I had walked over there Dr. Prichard arrived, and drove me back in his carriage. He asked me what Latin and Greek we were doing at the College. I mentioned amongst others the *Clouds* of Aristophanes, and referred to the very long words it contained. He asked me to quote one, and I gave σφρᾱγῖδ-ονυχ-αργο-κομήτης. He made me give him all the derivations of this compound word, and when I came to grief over one he corrected me, and quoted not only many other of the long words, but also several passages from the comedy. In fact, he seemed to know the whole play by heart.

"It must have been to him a profound and lasting pleasure to contemplate his large family growing up examples of conduct which their preceptor Dr. Jerrard would have described as 'Perfectly unexceptionable.' Although he had to suffer the inexpressible grief of losing some of his sons early in life, yet he must have had the great consolation of knowing how much of those lives, alas! too short, had been devoted to the service of God and the good of mankind.

"Not least of them in usefulness was Augustin, who after a life of nearly eighty years has left the world richer than he found it, and by his consistent rectitude, his honourable relation towards all those with whom he was brought in contact, and his devotion to his special work, has left to younger men an example which they will do well to follow."

Mr. ALEXANDER WAUGH writes:

"All who knew Augustin Prichard have sustained a great loss by his death. He possessed an unrivalled personality and a tenderness of heart, combined with great force of character and determination, which could only be fully realised by a long and personal intimacy.

"I remember, as if it was only yesterday, when I first saw him in the autumn of 1859, the occasion being an interview previous to the signing of the apprenticeship deeds which bound me to him for three years. I remember the awe inspired by meeting the great surgeon, which was not lessened by the seemingly stern manner with which he received me. On handing him my testimonials from college, he remarked: 'I shall judge myself; if you work well I will look after you, if you don't I shall not.'

"As a master he was most particular as to punctuality, and I think I see him now striding down Park Street every Sunday morning on his way to the Eye Dispensary, which he reached punctually as the clock struck nine, and looked back to see if I were close at hand. He was particularly attracted to ophthalmology, and it was a treat to see him operate on double cataract, with his right and left hand alternately, and the results were persistently good.

"As a dresser under him for three years, I had ample opportunity of seeing his work. He was great on dressings after operations, and enveloped a stump in numerous layers, always commencing with the simple dressing ointment 'spread thick.' In 1860 he began what was really the commencement of antiseptic surgery by dressing all wounds with the compound tincture of benzoin, and the results from this treatment were very good, especially in lacerated wounds of the hand. I carried out for him the treatment of carbuncle with potassa fusa, and the results were most satisfactory. The part was frozen first with a mixture of ice and salt, and then the potash was applied freely and made a slough, according to the size of the patch. He made this treatment of carbuncle the substance of his address in surgery at the annual meeting of the British Medical Association in Bristol, in 1863. He was a very successful operator in stone cases. After its introduction he invariably performed Allarton's operation, and invented the straps for this operation.

"Apart from his profession, he was a very well read man, a good classical scholar, and an excellent draughtsman. In politics he was a staunch Conservative, and never missed the dinner of his party on Colston Day. As a friend he was most faithful, and it is impossible to think of him as an enemy to anyone. As a consultant he was the soul of honour, and it was always a great pleasure and privilege to be associated with him in the treatment of a case. His opinion was always a decided one, and his manner commanded respect and faith."

Mr. Charles Randolph writes:

"I was one of Mr. Prichard's last dressers at the Bristol Royal Infirmary, and very sorry we were when the twenty years' service came to an end and we had to say good-bye to our old master. As a parting present he gave us each a copy of his *Ten Years of Operative Surgery*, a book full of interest, and from which I have since derived much practical information. We were all fond of Prichard, and used to wait for him in the lobby outside the Porter's room, and when the pair of black horses, driven by his old coachman, himself a well-known character, deposited their master at the foot of the steps, we were always glad to see him.

"He always greeted his students with a silent nod, and a wonderful nod was that nod of Prichard's—it carried with it a fatherly feeling that words could not express. He was a man of few words, never irritable, very kind to all with whom he had to do, free from all fads, thoroughly practical in his teaching and practice, and beloved by students and patients alike.

"Had there been a capital operation the day before, or any patient about whom he was more than usually anxious, his first words were (calling the dresser who had charge of the case): 'So-and-so, how is that man?' For I believe he had as much affection for every hospital patient, and suffered as much personal anxiety on their behalf as he did for his best private patient. He always had a cool head and a steady hand, and was a very careful operator, though when circumstances required it he would finish an operation with great rapidity. He was generally anxious to avoid the prolonged administration of chloroform. I do not know whether he ever had an accident in his surgical practice with anæsthetics, but I know he did not love them very much, and I have seen him cast many an enquiring glance at the patient to satisfy himself that all was right."

Dr. E. Long Fox writes:

"My intimacy with Mr. Augustin Prichard began more than forty years ago. He was then quite the leading surgeon and the best operator in Bristol, and had a deservedly high reputation in the treatment of diseases of the eye. Associated with him all these years on the Infirmary staff, I can speak of him as a most affectionate friend, wise and kind and tender. For twenty years we worked together as colleagues in caring for the health of Clifton College, and no institution in England ever had a more efficient surgeon than he was. Others will speak of his operations as surgeon with more effect than I can; but I have often seen him at work, and his skill was only equalled by his extreme care that his patients should have every chance of recovery.

"He was especially a strong colleague, devoted to his work, with a very real love for the Infirmary, anxious to keep the medical school at a high level, learned in many directions, taking a high view of life, and of his duty in life.

"His tenderness to children, and the confidence they had in him, was a thing to see; and as I lived close to him for some years, there were daily opportunities of seeing how he encouraged and even shared the fun of his family.

"He was perhaps rather a shy man; but those whom he allowed to know him well found personified in his life, duty, honour, skill, and a sweet homely kindliness based on the deepest foundations."

Dr. W. J. Fyffe writes:

"In September, 1882, I was appointed Physician to Clifton College. At that time Mr. Prichard had been twenty years surgeon to the school, and I shall always remember with gratitude the cordial assistance he gave me on my first undertaking duties which were entirely new to me.

"His long experience of life, his sound judgment and strong common-sense, and his deep loyalty to the College which he had seen growing with marvellous strides, made Mr. Prichard more than a professional colleague — I found him to be a tower of strength at every turn.

"In every difficult or anxious case occurring in the school he was ever ready to give me the assistance of his rich and matured experience; for although his great reputation was built mainly, I suppose, on his profound surgical knowledge, he was equally at home as a consultant in medical cases.

"But it was not alone in professional questions that Mr. Prichard proved himself to be so valuable a colleague. It was evidenced still more in the strength of his personality. The tone of his character, his high sense of honour and morality, made him a man to be relied on in any matter of doubt or difficulty, while his genuine kindness of heart, his sympathy with the young and ardent life with which we were surrounded, coupled with his long experience and knowledge, established that confidence between himself and the members of the school and its governing authorities which made the wheels of our professional life in Clifton College run smoothly and without friction. He retired from this branch of his work in 1890, carrying with him the respect and regard of all connected with the College which he had faithfully served for thirty years.

"His professional intercourse with me ripened into a warm friendship, which terminated only with his life."

### BIBLIOGRAPHY OF THE WORK OF AUGUSTIN PRICHARD.

On the crania of the Laplanders and Finlanders, with observations on the differences they presented from other European races. *Proc. Zool. Soc. Lond.*, 1844, xii. 129—135.

The Bristol Medical School. *Lancet*, 1846, i. 258.

Case of cataract occurring in early life. *Prov. M. & S. J.*, 1847, 539—541.

Translation of Vol. II. of Alexander von Humboldt's Κόσμος: *A general survey of the physical phenomena of the universe.* Hippolyte Baillière. London. 1848.

Coroners' inquests. *Prov. M. & S. J.*, 1848, 502-3.

Surgical cases at the Bristol Royal Infirmary. *Ibid.*, 1851, 66—67, 348—350, 657—659.

Critical remarks on Mr. Skey's account of the peritoneal covering of the distended bladder. *Ibid.*, 194-5.

On the use of kousso. *Ibid.*, 1852, 51.

Extraneous substances in the eye. *Ibid.*, 582—584.

The address in surgery at the annual meeting of the British Medical Association in Swansea, 1853. [Not published.] See *Assoc. M. J.*, 1853, 737; also Sir Charles Hastings' Address, *Ibid.*, 1854, 973; also Mr. Prichard's letter, *Ibid.*, 1045.

Tumour in the groin, with symptoms of strangulated hernia: operation: death. *Ibid.*, 1076.

Fatal injuries of the head in children. *Ibid.*, 1855, 318.

Case of lithotomy presenting unusual difficulties, and one of staphyloraphy. *Ibid.*, 1051-2.

Pistol-shot wound of the chest: wound of the heart and stomach. *Ibid.*, 1085-6.

Secondary hæmorrhage after amputation. *Ibid.*, 1145.

Case of aneurism of the common femoral artery treated successfully by compression: with remarks. *Ibid.*, 1856, 4.

Autoplastic surgery of the eyelids. *Ibid.*, 104—106.

The operation for the extraction of cataract. *Ibid.*, 365-6, 415.

The treatment of tetanus: with cases. *Ibid.*, 473-4.

Compound fracture of the thigh, and primary and secondary amputation. *Ibid.*, 753—755.

Two cases of removal of extraneous substances from mucous cavities. *Ibid.*, 863-4.

The late fatal chloroform case. *Ibid.*, 945.

The anatomy, physiology, and diseases of the membrana pupillaris. *Brit. M. J.*, 1857, 339—342.

Speculum cases. *Ibid.*, 513.

Presidential Address to the Bath and Bristol Branch of the British Medical Association. *Ibid.*, 629—632.

Lithotomy:—Three cases of stone in the bladder, in which Allarton's operation was performed.[1] *Ibid.*, 727-8.

Cases of strangulated hernia requiring operation. *Ibid.*, 899—901.

Diphtheritic conjunctivitis. *Ibid.*, 981—983.

Death from chloroform. *Ibid.*, 1858, 207-8, 252.

Case of intussusception: surviving five months. *Ibid.*, 264-5.

Lithotomy. *Ibid.*, 387—389.

Strangulated umbilical hernia in a male subject: operation : recovery. *Ibid.*, 901-2.

Sympathetic irritation of one eye. *Med. Times & Gaz.*, 1859, i. 351.

Cases of extirpation of the eye. *Brit. M. J.*, 1859, 328—330.

The median operation of lithotomy for the removal of a portion of bougie from the bladder.[1] *Ibid.*, 351-2.

Nyctalopia. *Ibid.*, 447-8.

Case of lumbricus. *Ibid.*, 854-5.

Amputation of the penis. *Ibid.*, 882.

Case of epilepsy. *Ibid.*, 1860, 319.

Ten years of operative surgery in the provinces. *Ibid.*, 774, 795, 818, 833, 856, 876, 893, 951, 972, 991; 1861, i. 10, 32, 62, 165, 383, 411, 518, 625; 1861, ii. 85, 168, 226; 1862, i. 304, 358, 408. (Also reprinted in book form. T. Richards. London. 1862.)

Case of popliteal aneurism cured by forcible flexion. *Ibid.*, 1861, i. 334-5.

Dressing of scalp-wounds. *Ibid.*, 593.

Lithotomy by Allarton's median operation.[1] *Ibid.*, 1862, i. 280—282.

Chlorodyne. *Ibid.*, 556-7.

On the immediate treatment of stricture of the urethra. *Ibid.*, 650-1.

Case of sphacelus of the tongue. *Ibid.*, 1862, ii. 487-8.

Wound of the vertebral artery. *Ibid.*, 1863, i. 399—400.

The address in surgery at the annual meeting of the British Medical Association in Bristol, 1863. [Carbuncle, and its treatment.] *Ibid.*, 1863, ii. 151—156. (Also reprinted in book form. T. Richards. London. 1863.)

Case of strangulated congenital hernia. *Ibid.*, 1864, ii. 728.

Lip-tourniquet. *Ibid.*, 1865, i. 222.

Gonorrhœal rheumatism. *Ibid.*, 1867, i. 382-3.

Ligature of the external iliac artery. *St. Barth. Hosp. Rep.*, 1867, iii. 227—234.

[1] These cases are also printed, with letters referring to them, in Allarton's *Median Lithotomy*, 1863, a book which was dedicated to Augustin Prichard and John Joseph Hadley "in acknowledgment of the important services they have rendered to the cause of humanity by their persistent and successful efforts to substitute Modern Median Lithotomy for the barbarous and fatal Operation in general use." Allarton was highly delighted with "the philosophic spirit displayed by the Bristol surgeons" in the consideration of his operation. He prints also letters and cases by Mr. Ralph M. Bernard, Mr. W. Michell Clarke, Mr. Thomas Green, Mr. John Harrison, and Mr. Henry A. Hore, all of whom were Bristol surgeons.

Case of antiphlogistic treatment. *Brit. M. J.*, 1868, i. 531-2.
A summary of surgical operations performed in the Bristol Royal Infirmary. *Ibid.*, 1870, i. 75-77.
On intraocular myotomy. *Ibid.*, 1871, ii. 578—580.
Chloroform accidents. *Ibid.*, 1873, i. 194—196.
Recurrent fibroid tumour of the breast [case]. *Tr. Bristol M.-Chir. Soc.*, 1878, i. 94—96.
Vesical calculus [case]. *Ibid.*, 96—7.
Obituary notice of Crosby Leonard. *Bristol Roy. Infirm. Rep.*, 1878-79, i. 342—360.
Obituary notice of William Budd. *Ibid.*, 361—367.
Multiple lipomata. *Brit. M. J.*, 1881, i. 271.
An address delivered at the opening of the section of surgery, at the annual meeting of the British Medical Association, held in Worcester, 1882. *Ibid.*, 1882, ii. 263—265; also *Med. Times & Gaz.*, 1882, ii. 233—235.
Address to the Students of the Bristol Medical School, at the Annual Distribution of Prizes. *Western Daily Press* and other Bristol papers of June 21st, 1884.
Rupture of the aorta. *Bristol M.-Chir. J.*, 1885, iii. 127—129.
The medical certificate of the cause of death. *Ibid.*, 1888, vi. 153—173.
The early history of the Bristol Medical School. *Ibid.*, 1892, x. 264—277.
One view of heredity. *Ibid.*, 1893, xi. 91—93.
Influenza in 1775. *Lancet*, 1894, i. 175-6.
*A few medical and surgical reminiscences.* 12mo. 99 pp., 1 portr. J. W. Arrowsmith. Bristol. 1896.
Intra-ocular myotomy. *Ann. Ophth.*, 1897, vi. 239—242.
An early prescription for cod liver oil. *Brit. M. J.*, 1897, ii. 52.

